# Primary cardiac mesothelioma presenting with fulminant recurrent pericarditis: a case report

**DOI:** 10.1093/ehjcr/ytad100

**Published:** 2023-02-24

**Authors:** Shmuel Schwartzenberg, Yaron Shapira, Victor Rubachevski, Ram Sharony

**Affiliations:** The Department of Cardiology, Rabin Medical Center, 39 Jabotinsky St., Petah Tikva 4941492, Israel; Affiliated with the Sackler Faculty of Medicine, Tel-Aviv University, Tel Aviv, Israel; The Department of Cardiology, Rabin Medical Center, 39 Jabotinsky St., Petah Tikva 4941492, Israel; Affiliated with the Sackler Faculty of Medicine, Tel-Aviv University, Tel Aviv, Israel; Affiliated with the Sackler Faculty of Medicine, Tel-Aviv University, Tel Aviv, Israel; The Department of Cardiothoracic Surgery, Rabin Medical Center, 39 Jabotinsky St., Petach Tikva 4941492, Israel; Affiliated with the Sackler Faculty of Medicine, Tel-Aviv University, Tel Aviv, Israel; The Department of Cardiothoracic Surgery, Rabin Medical Center, 39 Jabotinsky St., Petach Tikva 4941492, Israel

**Keywords:** Case report, Recurrent pericarditis, Pericardial mesothelioma, Transthoracic echocardiogram

## Abstract

**Background:**

Primary pericardial mesothelioma is an extremely rare disease. Prognosis is poor, with little effects of chemo- or radio-therapy. The majority of cases is diagnosed at autopsy.

**Case summary:**

A 22-year-old man, who presented with recurrent pericarditis and large pericardial effusion 2 months after a second BNT162b2 COVID-19 vaccine, underwent pericardiocentesis and pericardial window. Pathology specimen of pericardium revealed benign mesothelial inflammation, consistent with acute pericarditis. Four months later, he presented with a large pericardial mass manifesting in heart failure and underwent urgent pericardiectomy. A new pathology specimen immunostaining and fluorescence *in situ* hybridization analysis revealed pericardial mesothelioma. Despite intensive care, the patient died 3 weeks later.

**Discussion:**

Primary pericardial mesothelial should be considered in the differential diagnosis of refractory recurrent pericarditis, even with prior biopsy-proven pericarditis or when a putative trigger (COVID-19 mRNA prior vaccination) is suspected, as was the case in this patient. Tumour diagnosis and identification consist of multimodal imaging and laboratory tests. A multidisciplinary, individualized care approach should be performed.

**Learning points**

- Alert the clinician to an unexpected manifestation of a rare cardiac malignancy.
- Extensive pericardial involvement by pericardial malignancy can mimic pericardial effusion on cardiac imaging.
- The diagnosis is challenging and requires a team approach and multimodal imaging.

## Introduction

Primary pericardial mesothelioma is an extremely rare disease that accounts for 0.8% of all mesotheliomas and 2–3% of all pericardial tumours.^1,2^ Prognosis is very poor, with little effects of chemo- or radio-therapy and median survival about 4–6 months.^3^ The majority of cases is diagnosed at autopsy,^4^ with anecdotal case reports of constrictive pericarditis, cardiac tamponade, and heart failure, as was the case in our patient.^5–7^ Cardiac imaging is non-specific: in a long-term Chinese medical literature database of 64 patients with primary pericardial mesothelioma, the most common echocardiographic presentations were pericardial effusion (85.9%), pericardial masses (36.4%), and pericardial thickening (17.3%).^8^ We hereby present a rare case of pathologically confirmed recurrent pericarditis that transformed into mesothelioma, within only 4 months.

## Timeline

**Table:** 

Date	Event
1 February 2021	• Patient receives second BNT162b2 COVID-19 vaccine
10 March 2021	• First episode of pericarditis with moderate pericardial effusion
	• Pericardiocentesis removal of 900 cc of sero-sanguineous exudative fluid
	• Discharged with ibuprofen and colchicine treatment
1 April 2021	• Readmitted with large pericardial effusion
	• Undergoes pericardiocentesis with drainage of 1 L prednisone 40 q.d., and colchicine 500 mg b.i.d. is initiated
9 April 2021	• Pericardial window with pathology specimen consistent with acute pericarditis
15 August 2021	• Readmitted with pleuritic chest pain and dyspnoea
	• Transthoracic echocardiogram and computed tomography show thick pericardial layer with constrictive physiology
18 August 2021	• Pericardiectomy with partial relief of constrictive layer
	• Veno-arterial extracorporeal membrane oxygenator support is instituted
	• Develops multiorgan failure
11 September 2021	• Patient died

## Case presentation

**Figure 1:**
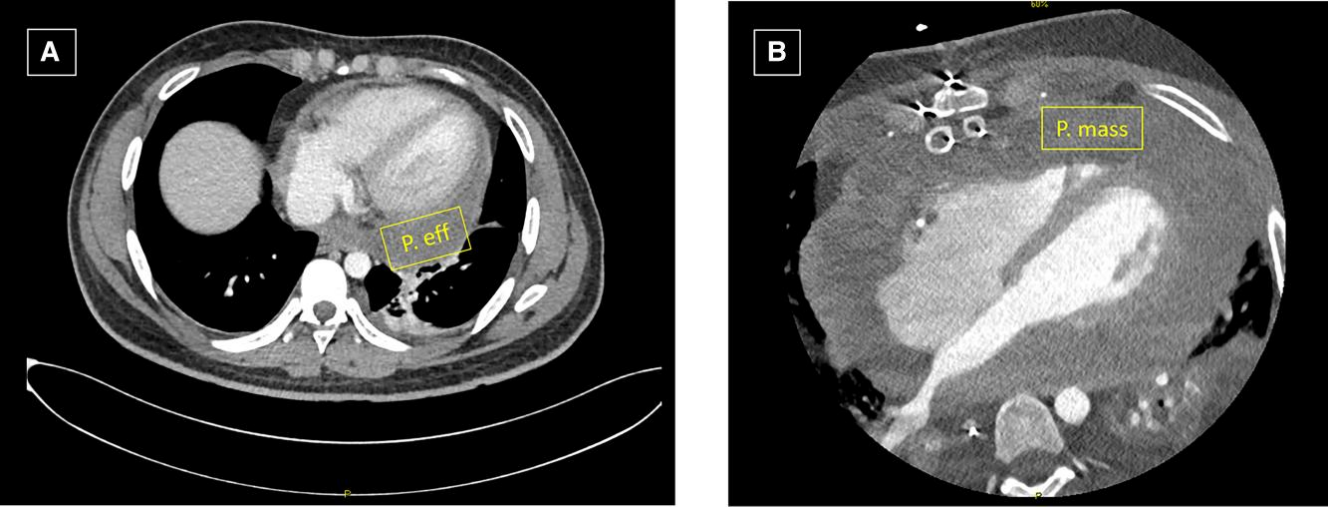
Chest computed tomography showing pericardial thickening with moderate pericardial effusion (P. eff) (*A*) and large pericardial mass (moreover) with compression of left atrium (LA) 4 months later (*B*).

A 22-year-old healthy male presented with a 4-day pleuritic chest pain and dyspnoea. There was no history of smoking or previous asbestos exposure. He had received his second BNT162b2 (Pfizer-BioNTech) vaccine a month earlier. Heart rate was 121 b.p.m. and blood pressure 144/95 mmHg. He was afebrile without abnormal auscultatory findings. Electrocardiogram showed diffuse ST elevation. He had mild leucocytosis (11 930 WBCs/µL). Echocardiography revealed moderate pericardial effusion with normal heart function. Acute pericarditis was diagnosed, and treatment with ibuprofen (400 mg t.i.d.) and colchicine (0.5 mg b.i.d.) was initiated. He remained symptomatic, and 2 days later underwent pericardiocentesis with drainage of 900 cc of sero-sanguineous exudative fluid with clinical improvement, following which he was discharged on non-steroidal anti-inflammatory drugs and colchicine. One month later, he developed recurrent similar symptoms and a large pericardial effusion. Repeat pericardiocentesis was performed with drainage of 1 L of fluid. Pericardial cytology was negative for malignant cells, bacterial culture, or acid-fast staining. Symptoms persisted despite prednisone 40 mg/day treatment. A cardiac computed tomography (CT) (*Figure 1A*) performed due to recurrent pericarditis demonstrated pericardial thickening with moderate pericardial effusion. Pericardial window was performed via left thoracotomy after multidisciplinary team discussion. Pathology specimen revealed fragments of pericardium showing benign acute and chronic inflammation with benign mesothelial proliferation. The patient was discharged uneventfully with prednisone 40 mg q.d. and colchicine 0.5 mg b.i.d.

Four months later, he was re-admitted with recurrent pleuritic chest pain and dyspnoea without fever. He was tachycardic 110 b.p.m. and had elevated jugular venous pressure but no pulsus paradoxus.

**Figure 2:**
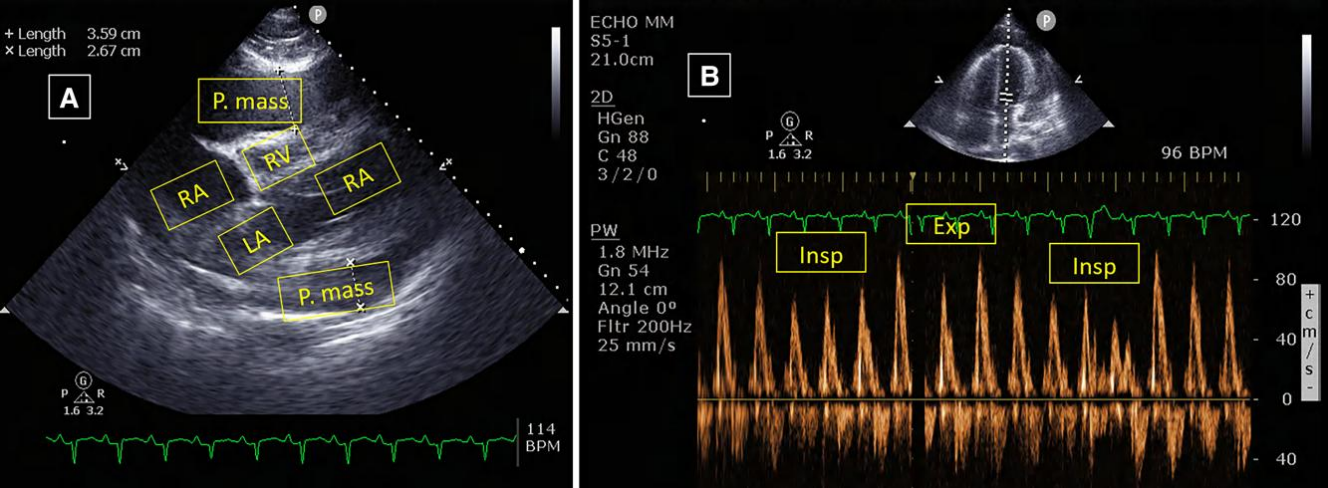
Transthoracic echocardiogram showing large pericardial mass/effusion compressing right ventricle (*A*) with significant respiratory mitral valve flow variation in E-wave velocity with respiration on pulse-wave Doppler (*B*). LV, left ventricle; RA, right atrium; RV, right ventricle.

Laboratory investigations showed normal routine chemistry including normal levels of troponin (<14 ng/mL) and creatinine phosphokinase 80 U/L (normal 20–200 U/L). C-reactive protein level was elevated at 5 mg/dL (normal 0–0.5 mg/dL). Four blood culture specimens were negative, and three nasopharyngeal swab COVID-19 PCR tests were negative. Serology for HIV, Rickettsia, Q fever, and cytomegalovirus were also negative. Chest X-ray revealed cardiomegaly, and electrocardiogram showed sinus rhythm 110 b.p.m. with lateral lead T-wave inversion. A new CT study (*Figure 1B*) revealed a large heterogenous pericardial layer of 4 cm thickness. Transthoracic echocardiogram (TTE) revealed right ventricle (RV) dysfunction and a large pericardial effusion with constrictive physiology (*Figure 2*).

**Figure 3:**
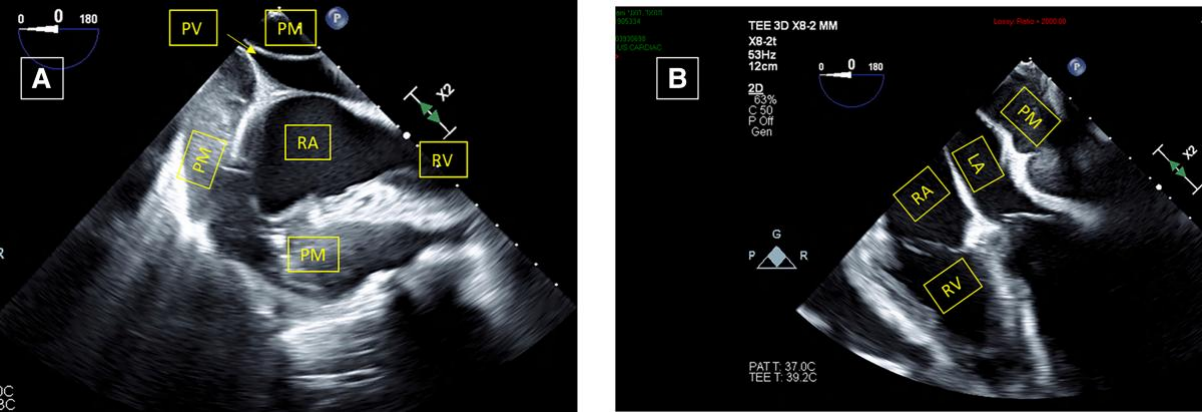
(*A*, *B*) Intraoperative transoesophageal echocardiography study showing large echogenic pericardial mass (PM) enwrapping right atrium (RA) and right ventricle (RV) and compressing right upper pulmonic vein (PV) and left atrium (LA).

**Figure 4:**
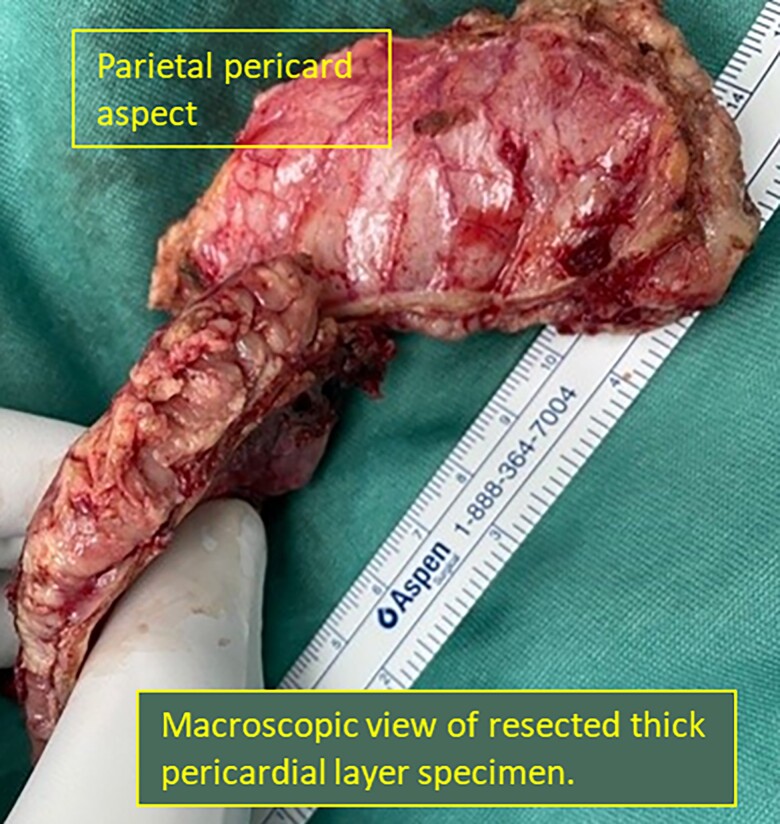
Macroscopic view of resected pericardial layer.

**Figure 5:**
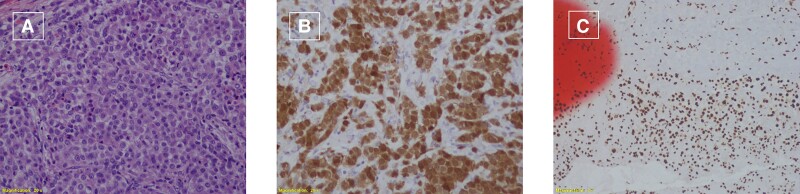
(*A*) Haematoxylin and eosin staining showing mitosis and necrosis of tumour cells. (*B*) Positive calretinin immunohistochemical staining. (*C*) Pathology BAP-1 positive stain performed on the specimen from the first operation when pericarditis was present.

He underwent exploratory thoracotomy and pericardiectomy. Intraoperative transoesophageal echocardiography (*Figure 3*) revealed a large echogenic pericardial mass enwrapping the heart and compressing the pulmonic veins and left atrium, with severe RV dysfunction. Upon mid-sternotomy, the entire heart and the great vessels were wrapped by a severely inflamed 3 cm thickness of fibro-gelatinous layer, without pericardial effusion. Only partial relief of the constrictive layer was technically achievable. The patient was then connected to veno-arterial extracorporeal membrane oxygenator (VA ECMO) circuit and transferred to the intensive care unit. After prednisone and colchicine treatment, he was switched to anakinra (pending pathology diagnosis from intraoperative specimen under a working hypothesis of refractory recurrent pericarditis), but he developed multiorgan failure. Transthoracic echocardiogram revealed persistence of the large pericardial mass and RV dysfunction. Repeat pericardiectomy was attempted, with partial piecemeal resection of the thick layer around the two venae cavae and right pulmonary veins (*Figure 4*). Unfortunately, despite inotropes, ECMO support, and continuous renal replacement therapy, the patient died 3 weeks later. Pathology specimen from operation (*Figure 5*) revealed epithelioid cells with irregular vesicular nuclei, palely eosinophilic cytoplasm, and frequent mitotic figures consistent with pericardial malignant mesothelioma. Immunostaining was strongly positive for all mesothelial markers including calretinin, KER7, D2-40, Glut1, and CK 5/6. Fluorescence *in situ* hybridization (FISH) analysis detected CDKN2A homozygous deletion in 70% of the cells, verifying the diagnosis of malignant mesothelioma.^9^

## Discussion

Our initial working hypothesis was that the patient had fulminant recurrent pericarditis complicated by pericardial tamponade. After an episode of acute pericarditis, the probability of developing incessant pericarditis or first recurrence within 18 months is 15–30%, with 25–50% likelihood of additional recurrence after first recurrence.^10,11^ The unusual manifestation of recurrent fulminant refractory pericarditis without evidence of any underlying medical condition prompted us to consider his COVID-19 vaccination as a putative risk factor in view of anecdotal reports at that time suggesting an increased risk of pericarditis in young adults after administration of the Pfizer-BioNTech mRNA-based vaccine.^12^ This was subsequently refuted in a large study in nationwide setting in Israel.^13^ In retrospect, the unusual fulminant presentation with constriction and particularly the operative findings should have alerted us before the pathology report was issued to the correct diagnosis of malignancy in the absence of infection or autoimmune aetiology.

In order to exclude the possibility that mesothelioma was missed in the first pathology specimen obtained at the time of pericardial window, a thorough pathologic examination was performed: no evidence of dysplasia or atypical changes were noticed. Moreover, a BRCA1-associated protein 1 (BAP-1) immunohistochemical stain was positive. The finding of loss of BAP-1 is 100% specific for malignant mesothelioma, and it has not been reported in benign mesothelial proliferation.^14,15^ As shown in *Figure 5C*, the BAP-1 stain was indeed positive in the first specimen, consistent with the diagnosis of pathologically confirmed pericarditis that transformed into mesothelioma, in this case within only 4 months. Lee *et al.*^16^ reported that indolent cases of well-differentiated papillary mesothelioma evolve as diffuse, widespread malignant mesothelioma 10 years after the initial diagnosis with BAP-1 loss, suggesting that BAP-1 loss might be a predictor of subsequent development of a malignant mesothelioma. The mechanism by which pericarditis transforms into mesothelioma is unknown. To the best of our knowledge, this is the only second case of pathologically confirmed pericarditis that transformed into mesothelioma,^17^ in this case within only 4 months. It is unlikely that an earlier correct diagnosis would have made a clinical difference in this case in view of the accelerated disease course.

## Conclusion

Primary pericardial mesothelioma should be considered in the differential diagnosis of refractory recurrent pericarditis, even with prior biopsy-proven pericarditis. Extensive pericardial involvement by the pericardial malignancy can mimic pericardial effusion on cardiac imaging, which can be particularly misleading if the patient did have also previously documented pericardial effusion, as illustrated in this case.

## Supplementary Material

ytad100_Supplementary_Data

## Lead author biography

Senior cardiologist at the Rabin Medical Center, where he works full time as an attending in the Echocardiography Lab and Valve Clinic and engages in related clinical research.

## Supplementary material

**Slide sets:** A fully edited slide set detailing this case and suitable for local presentation is available online as Supplementary data.

**Consent:** The authors confirm that written consent for submission and publication of this case report including images and associated text has been obtained from the patient in line with COPE guidance.

**Funding:** None declared.
